# Effects of intensive repetition of a new facilitation technique on motor functional recovery of the hemiplegic upper limb and hand

**DOI:** 10.3109/02699052.2010.506855

**Published:** 2010-08-17

**Authors:** Kazumi Kawahira, Megumi Shimodozono, Seiji Etoh, Katsuya Kamada, Tomokazu Noma, Nobuyuki Tanaka

**Affiliations:** 1 Department of Rehabilitation and Physical Medicine, Kagoshima University Graduate School of Medical and Dental Sciences, Kagoshima, Japan; 2 Kagoshima University Hospital Kirishima Rehabilitation Center, Kagoshima, Japan

**Keywords:** Facilitation technique, hand, hemiplegia, repetitive facilitation exercise, stroke, upper limb

## Abstract

**Objective:**

To study the effects on the hemiplegic upper limb of repetitive facilitation exercises (RFEs) using a novel facilitation technique, in which the patient's intention to move the hemiplegic upper limb or finger was followed by realization of the movement using multiple sensory stimulations.

**Methods:**

Twenty-three stroke patients were enrolled in a cross-over study in which 2-week RFE sessions (100 repetitions each of five-to-eight types of facilitation exercise per day) were alternated with 2-week conventional rehabilitation (CR) sessions, for a total of four sessions. Treatments were begun with the 2-week RFE session in one group and the 2-week CR session in the second group.

**Results:**

After the first 2-week RFE session, both groups showed improvements in the Brunnstrom stages of the upper limb and the hand, in contrast to the small improvements observed during the first CR session. The Simple Test for Evaluating Hand Function (STEF) score, which evaluates the ability of manipulating objects, in both groups improved during both sessions. After the second 2-week RFE and CR sessions, both groups showed little further improvement except in the STEF score.

**Conclusion:**

The novel RFEs promoted the functional recovery of the hemiplegic upper limb and hand to a greater extent than the CR sessions.

## Introduction

Various physiotherapies have been developed to improve functional recovery in patients with a hemiplegic upper limb due to stroke or acquired brain injury, including the facilitation technique with proprioceptive neuromuscular facilitation [1], the Brunnstrom approach [2], the Bobath approach [3], electromyography (EMG)-initiated electrical stimulation [4], increased intensity physiotherapy [5], constraint-induced movement therapy [6, 7], computerized arm training [8], early and repetitive sensorimotor stimulation of the arm [9], transcranial magnetic stimulation (TMS) [10] and thermal intervention for the hemiplegic upper limb to facilitate sensory and motor recovery [11]. The standard neurophysiological facilitation techniques used for hemiplegic upper limbs have not been confirmed to promote the functional recovery of hemiplegic limbs [12–15].

**Figure 1:**
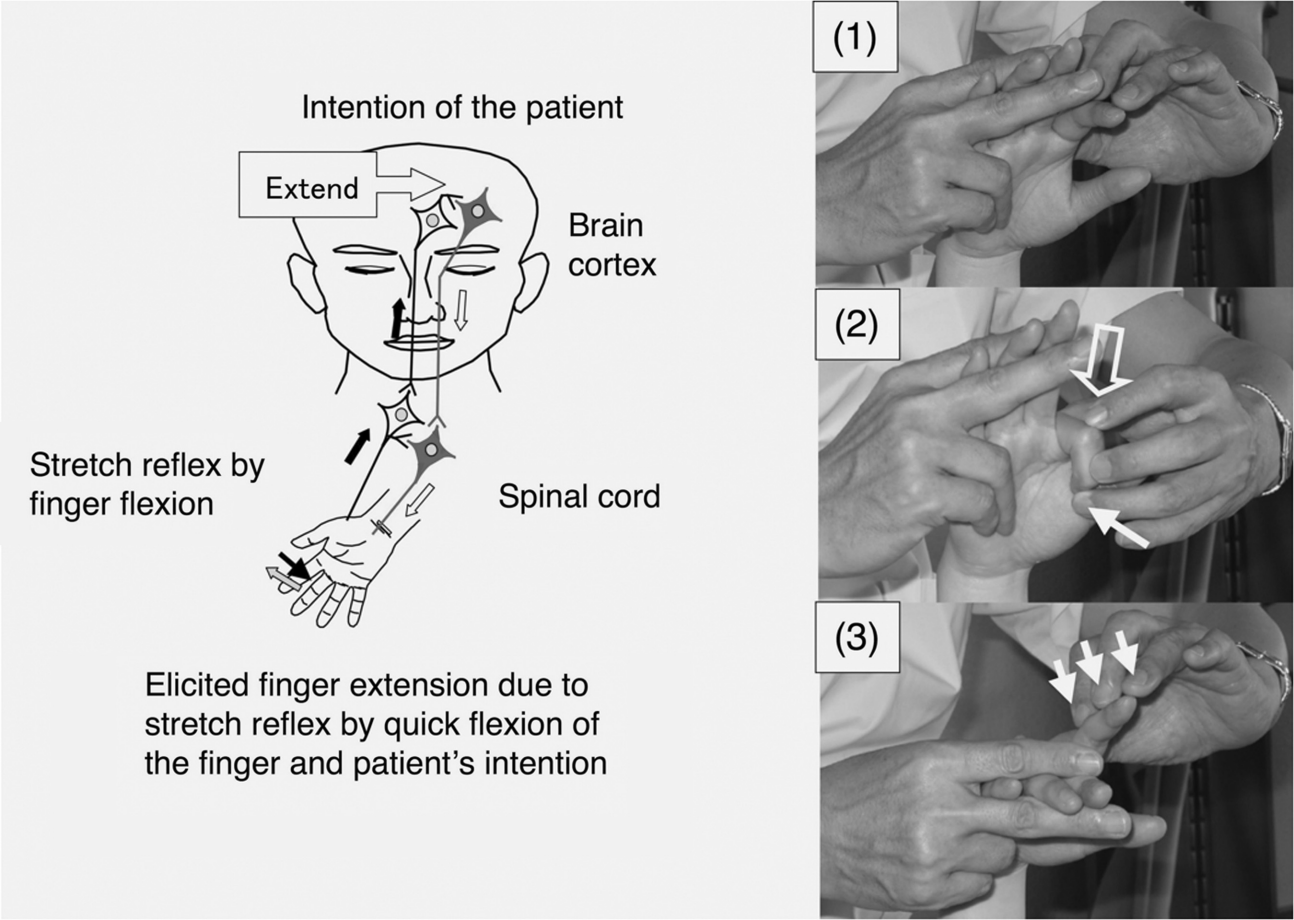
Hypothesized mechanism of action of a novel facilitation method for hemiplegic upper limb and fingers. (Left) The patient can realize his/her intended movements when neurons related to the intended movements are activated by the stretch reflex and neuronal excitation of the patient's intention comes from the prefrontal/premotor cortex. (Right) To facilitate extension of the isolated finger, it was quickly flexed by the therapist (1), the MP joint was flexed by the therapist after saying the instruction ‘Extend’ (2) and slight resistance against finger extension was applied during extension of the finger (3). The open arrow and closed arrow indicate manipulation of the stretch reflex and light touch (resistance) to maintain the α–γ linkage, respectively.

Previous studies on the efficacy of these neurophysiological approaches in promoting the functional recovery of hemiplegia have been limited by the small numbers of patients included, which have reduced the chance of detecting statistically significant differences, and by the use of measures that are not sufficiently sensitive to detect small improvements in motor function. In addition to the limitations associated with study design, these neu-rophysiological approaches mainly aim to normalize muscle tone or asymmetric posture and not to strengthen neuronal circuits through the injured descending motor tracts by repetition of the patient's intended movements [16]. The repetitive facilitation exercises (RFEs) using these novel facilitation methods for the upper limb and fingers, as shown in Figure 1, give sufficient physical stimulation, such as by the stretch reflex or skin-muscle reflex that is elicited immediately before or at the same time as when the patient makes an effort to move his hemiplegic hand or finger, in order to elevate the level of excitation of the corresponding injured descending motor tracts and it allows the patient to initiate movements of the hemiplegic hand or finger in response to his intention. The functional improvements seen with repetitive training of finger flexion and extension for the hemiparetic hand [17] and with RFEs for the hemiplegic lower limb [18] and for limb kinetic apraxia of the upper limb and fingers [19] have suggested that functional recovery of the hemiplegic upper limb and hand might depend on repetition of voluntary movements elicited by the RFEs, especially when they are influenced by a synergic pattern.

However, to the authors' knowledge, no previous studies have examined the effects of intensive repetition of facilitated movements isolated from the synergy of the hemiplegic upper limb and hand, because the existing facilitation techniques [1–3] are not suitable for repetitive facilitation such as 100 repeats of each isolated movement of the upper limb and each finger.

The present study investigated whether RFEs using the novel facilitation technique improved isolation from synergy and ability of manipulating the objects in the hemiplegic upper limb of patients with stroke.

## Methods

### Subjects

A total of 178 patients were admitted to the rehabilitation ward of the hospital for stroke rehabilitation between February 2001 and November 2002. Patients with the following characteristics were excluded from the present study: severe hemiplegia with a Brunnstrom stage (BRS) of 2 or less in the upper limb and hand; a medical or neurological condition that limited the effects of the RFEs, such as severe sensory disturbance, pain and contracture of the upper limb, severe aphasia that made it impossible to follow the verbal instructions of the therapist and dementia or visuo-spatial hemineglect that interfered with the outcome assessments or limited the patient's attention span or learning capacity; and failure to consent to participate.

The eligible subjects comprised 23 inpatients with hemiplegia due to stroke. The age and duration after onset were 54.7± 13.1 years (mean±SD; range, 31–81 years) and 15.6± 16.8 weeks (5–71 weeks), respectively. The BRS of the hemiplegic upper limb and hand was 4 (3–5) (median, quartiles) and 4 (3–5), respectively (Table I). Of the 103 patients who were admitted from February 2001 to February 2002, 12 were enrolled in the study and were initially treated with 2 weeks of conventional rehabilitation (CR) that did not include RFE (RFE group 2). Of the 75 patients who were admitted from March 2002 to November 2002, 11 were enrolled in the study and were initially treated with 2 weeks of CR that included RFE (RFE group 1).

**Table I:** Characteristics of the subjects with hemiplegia.

Group	RFE group 1 (*n* = 11)	RFE group 2 (*n* = 12)	All subjects (*n* = 23)
Age (years)	53.6±15.6 (31–81)	55.7±11.1 (41–73)	54.7±13.1 (31–81)
Gender			
Male/female	7/4	10/2	16/6
Diagnosis			
Haemorrhage/infarction	6/5	2/10	8/15
Site of lesion			
Putamen	3	5	8
Internal capsule/corona radiata	8	7	15
Side of hemiplegia			
Right/left	6/5	8/4	14/9
Time since onset (weeks)	15.5±19.0 (5–71)	15.7±15.5 (5–60)	15.6±16.8 (5–71)
Motor function (BRS)			
Upper limb	4.0, 3.0–5.0 (3–5)	4.0, 3.0–4.5 (3–5)	4.0, 3.0–5.0 (3–5)
Hand	4.0, 3.3–5.0 (2–5)	4.0, 3.0–5.0 (2–5)	4.0, 3.0–5.0 (2–5)

In RFE group 1, RFE was administered in weeks 1, 2, 5 and 6, while non-RFE was administered in weeks 3, 4, 7 and 8. In RFE group 2, non-RFE was administered in weeks 1, 2, 5 and 6, while RFE was administered in weeks 3, 4, 7 and 8.

RFE, repetitive facilitation exercise; CR, conventional rehabilitation; BRS, Brunnstrom stage.

All data are presented as the mean±standard deviation (range) or the median and quartiles (range).

This study was approved by the institutional ethical review board. All of the subjects provided informed consent.

### Exercise protocol

Two cross-over design studies were performed across individuals: A–B–A–B and B–A–B–A (where A denotes CR and RFEs and B denotes CR alone). The RFE sessions used a novel facilitation technique to elicit movements of the shoulder, elbow and each finger isolated from synergy. A 2-week RFE session was alternated with a 2-week CR session. Patients in RFE group 1 performed RFE sessions during weeks 1, 2, 5 and 6 and CR sessions during weeks 3, 4, 7 and 8 after admission to the hospital. Patients in RFE group 2 performed CR sessions during weeks 1 2, 5 and 6 and RFE sessions during weeks 3, 4, 7 and 8. The CR session consisted of range-of-motion exercises (ROMex) for the upper limbs, activities of daily living (ADL) training and occupational therapy including wiping, pinching and sanding movements of the hemiplegic upper limb with the assistance of an occupational therapist for 40min. The duration of the RFE session at 40 minutes was the same as that of the CR session. The ROMex for the upper limb and occupational therapy in the CR session were substituted by RFEs, which included the full range of motion of the joint of the upper limb in active or passive movements and were completed within 30 minutes, in the RFE session.

Each RFE session included between five and eight specific exercise patterns from a total of eight that were developed based on the novel facilitation technique including movement of each isolated finger. The novel facilitation technique involved the stretch reflex by rapid passive stretching of the muscles and the skin-muscle reflex which was induced by tapping or rubbing on the muscles in a specific posture that maintained the tension in the targeted muscles and tendons. The exercise patterns in an RFE session were changed as the hemiplegia improved. Additionally, each patient performed voluntary training freely without the assistance of a physical therapist or an occupational therapist for 1–3 hours. The patients underwent the RFE or CR sessions once a day for 5 days a week.

**Figure 2:**
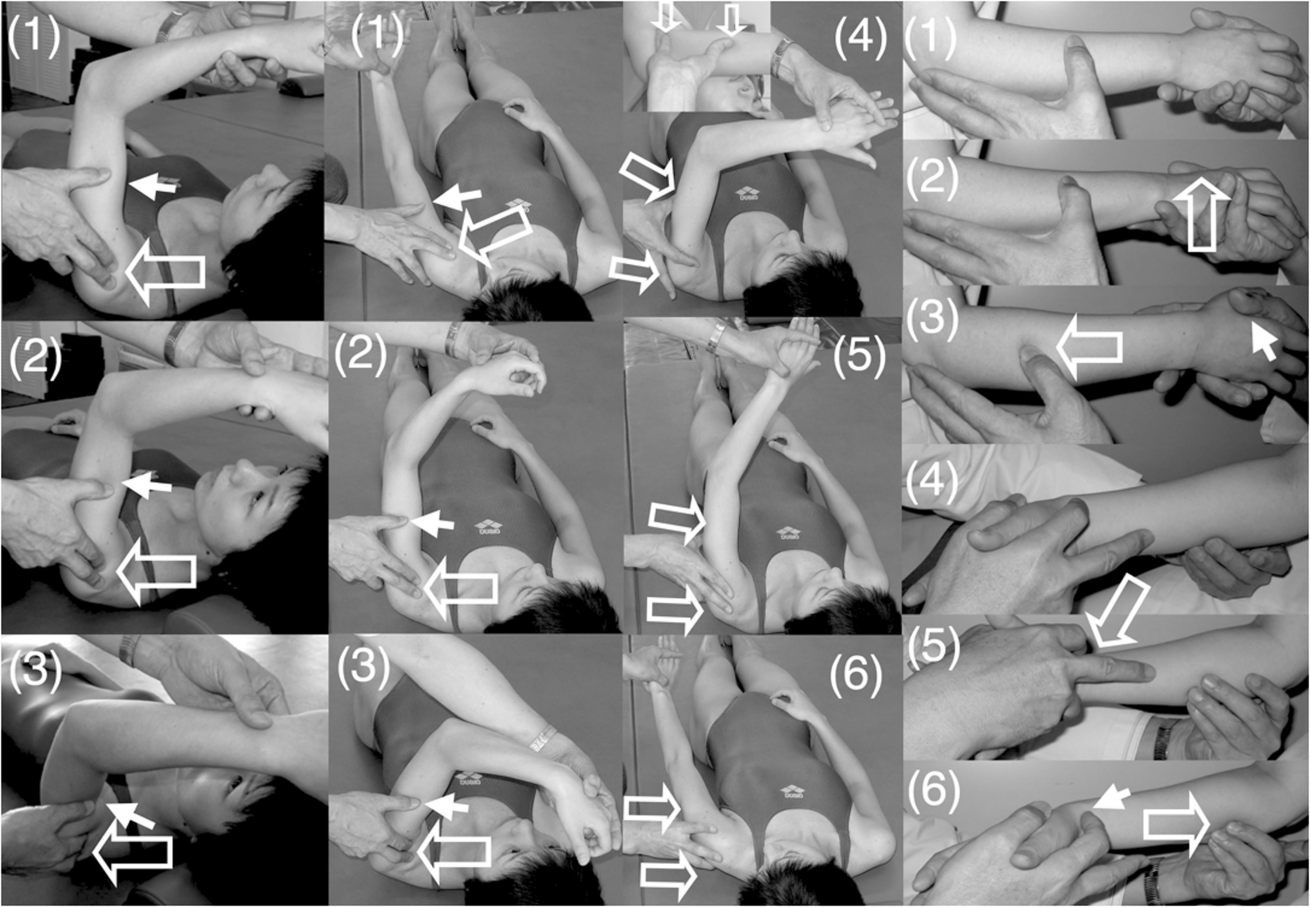
Shoulder flexion, shoulder adduction/flexion/internal rotation (modified PNF) and forearm supination/pronation with elbow flexion. (Left column) To facilitate shoulder flexion, the therapist tapped the anterior part of the deltoid muscle (1) and pushed the skin on the humeral head with his fingers to avoid its elevation (2) and supported or resisted the brachium by his thumb (3). (Middle two columns) To facilitate shoulder flexion/adduction/external rotation with flexing of the elbow and forearm supination accompanied by wrist flexion and finger flexion, the therapist quickly performed shoulder extension/abduction/internal rotation with extension of the elbow and forearm pronation accompanied by wrist dorsiflexion and finger extension (1), tapped the inside of the deltoid muscle (2) and provided support or resistance with his other hand (3). When the patient had achieved shoulder flexion/adduction/external rotation with flexion of the elbow, forearm supination accompanied by wrist flexion and finger flexion, the therapist quickly flexed the patient's wrist, pushed the tricepus blaki muscle and its tendon with the therapist's fingers to elicit elbow extension (4) and provided support by the therapist's other hand to the movements of shoulder extension/abduction/internal rotation (5), elbow extension/forearm pronation/wrist dorsiflexion/finger extension (6). (Right column) To facilitate forearm supination/pronation with 90° elbow flexion in the sitting position, the therapist held the hand of the patient and placed the thumb of his other hand on the dorsal forearm (1), quickly pronated the forearm (2), rubbed the dorsal forearm with his thumb and provided slight resistance by his hand (3). To facilitate forearm pronation, the therapist held the hand of the patient (4), tapped with his second finger the radial wrist for quick supination of the forearm (5) and rubbed the ventral forearm using his third and fourth fingers (6). The open arrow and closed arrow indicate manipulation of the stretch reflex and light touch (resistance) to maintain the α–γ linkage, respectively.

**Figure 3:**
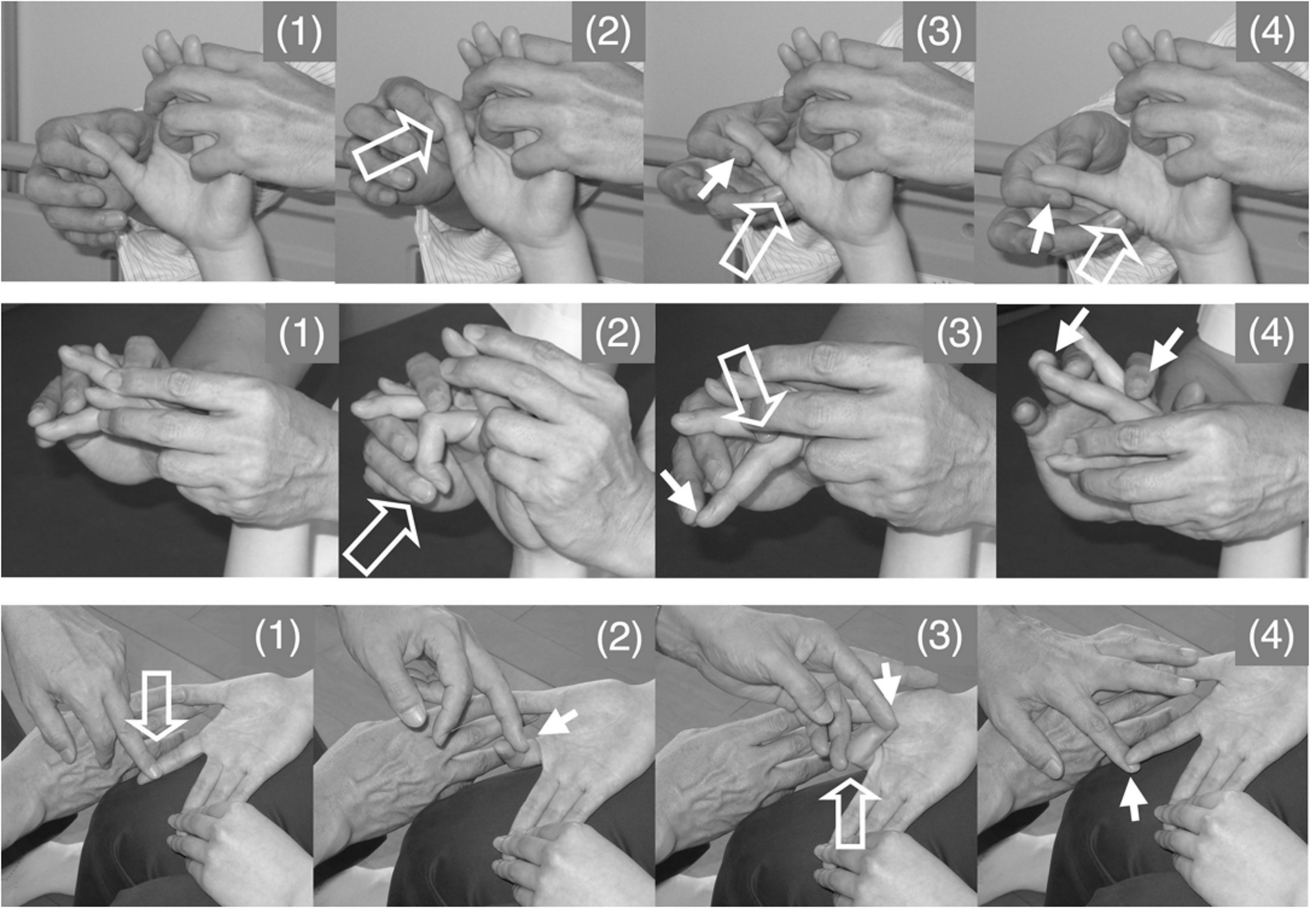
Volar abduction of the thumb, extension and extension/flexion of an isolated finger. (Upper row) To facilitate volar abduction of the thumb, the therapist held the thumb and second-to-fifth fingers of the patient (1), quickly pulled the thumb to achieve volar adduction (2), quickly tapped the radial side of the abductor pollicis brevis (3) and rubbed the muscle and applied slight resistance against finger volar abduction (4). (Middle row) To facilitate isolated extension of the middle finger, the therapist held the hand of the patient while keeping the patient's wrist flexed and fingers extended using his hands, placed his fingers on the MP joint, IP joint and nail of the middle finger (1), quickly pushed the nail to flex the middle finger (2), quickly pushed the finger proximal to the MP joint (3) and gave slight resistance against extension (4). (Lower row) To facilitate flexion/extension of the isolated finger, the therapist held the hand of the patient on the femur. The neighbouring fingers on both sides of the finger to be facilitated were held by the fingers of the therapist and by the patient. To facilitate flexion, the therapist quickly rubbed the finger from the proximal to distal position (1) and applied slight resistance against flexion (2). To facilitate extension of the finger, the therapist flexed the finger by the therapist's finger immediately after active finger flexion, flexed the MP joint (3) and applied slight resistance against finger extension (4). The open arrow and closed arrow indicate manipulation of the stretch reflex and light touch (resistance) to maintain the α–γ linkage, respectively.

The procedures of the eight new facilitation methods for the hemiplegic upper limb and fingers were as follows:

1. Shoulder flexion with 90° elbow flexion in the supine position (Figure 2, left column). When the therapist said ‘Flex’ the patient attempted to flex the hemiplegic shoulder. Then, to facilitate shoulder flexion, the therapist tapped the anterior part of the deltoid muscle with his fingers and then pushed on the humeral head to avoid impingement in the shoulder.
2. Shoulder horizontal extension/flexion with elbow flexion in the supine position. When the therapist said ‘Extend’ or ‘Flex’ the patient attempted to extend or flex his shoulder, respectively. To facilitate shoulder horizontal extension/flexion, rapid stretching and rubbing of the deltoid muscle were applied by the therapist.
3. Shoulder flexion/adduction/external rotation with flexion of the elbow and forearm supination accompanied by wrist flexion, finger flexion and shoulder extension/abduction/internal rotation while extending the elbow and pronating the forearm accompanied by wrist dorsiflexion and finger extension in the supine position (Figure 2, two middle columns; modified PNF). When the therapist said ‘Hold my hand and carry it to the top of your head’, the patient attempted to perform this movement, which involves shoulder flexion/adduction/external rotation. When the therapist said, ‘Extend your fingers and push my hand to the side of your torso’, the patient attempted to perform this movement, which involves shoulder extension/abduction/internal rotation. To facilitate the movements, tapping, rubbing and rapid stretching of the muscles were applied by the therapist.
4. Shoulder flexion/abduction/external rotation with elbow extension accompanied by wrist dorsiflexion and finger extension (modified PNF). When the therapist said, ‘Raise your hand over your head as if you were wiping your face with your forearm’, the patient attempted to perform this movement, which involves shoulder flexion/abduction/external rotation. The therapist used his hand to hold the patient's upper limb in a posture of shoulder extension/ adduction/internal rotation, elbow extension and forearm pronation. The therapist then quickly pulled the patient's upper limb to achieve shoulder extension/adduction/internal rotation and tapped and rubbed the inside of the deltoid muscle using his fingers to elicit shoulder flexion, while his thumb provided resistance to facilitate the shoulder external rotation. During these movements, the therapist supported the patient's arm with his other hand.
5. Forearm supination/pronation with 90° elbow flexion in the sitting position (Figure 2, right column). When the therapist said ‘Turn your hand (palm) upward’, the patient attempted to perform forearm supination and when the therapist said, ‘Turn your hand (palm) down ward’, the patient attempted to perform forearm pronation. To facilitate the movements, tapping, rubbing and rapid stretching of the muscles were applied by the therapist.
6. Wrist dorsiflexion and forearm pronation with extension of the fingers in the supine position. When the therapist said ‘Turn your forearm as if you were fanning wind to your face with the back of your hand’ or ‘Turn your forearm and hand as if you were fanning wind to your face with the back of your hand’, the patient attempted to perform wrist dorsiflexion and forearm pronation. The therapist held the abductor pollicis brevis in his hand and held fingers two-to-five in the wrist flexion position using the second and third fingers of his other hand. To facilitate forearm pronation and wrist dorsiflexion with finger extension, the therapist held the abductor pollicis brevis, quickly pulled the fingers, quickly supinated the forearm and tapped the ulnal side of the dorsal hand using his thumb. When the patient began to show wrist dorsiflexion and forearm pronation with extension of the fingers of his other hand, the therapist provided slight resistance to the patient's hand using his thumb and fingers.
7. Finger extension with wrist flexion in the supine position. When the therapist said ‘Extend’, the patient attempted to extend his finger. This exercise was performed by each of the five fingers of the hemiplegic hand (Figure 1, right, and Figure 3, upper and middle rows). To facilitate isolated volar abduction of the thumb, tapping, rubbing and rapid stretching of the muscles were applied by the therapist.
8. Finger extension/flexion with wrist flexion in the sitting position (Figure 3, lower row). When the therapist said ‘Flex your finger’, the patient attempted to flex his finger and when the therapist said ‘Extend your finger’, the patient attempted to extend his finger. To facilitate isolated finger extension/flexion, tapping, rubbing, rapid stretching of the muscles and slight resistance against finger movements were applied by the therapist.

Neural block and electrical treatments were not administered during the study period. The dose of muscle relaxant was not changed during the study period.

### Assessment of isolation from synergy and ability of manipulating the objects of the hemiplegic upper limb and hand

Isolation from synergy and ability of manipulating the objects of the hemiplegic upper limb and hand was assessed at 2-week intervals after either admission or the start of the experiments (that is, at weeks 0, 2, 4, 6 and 8) using the BRS for the upper limb and hand. A BRS of 6 is the highest score and represents normal movement. Ability or performance of manipulating the objects by the hemiplegic upper limb and hand was evaluated using The Simple Test for Evaluating Hand Function [19, 20]. The STEF was designed to evaluate the speed of carrying objects (three kinds of spheres, two kinds of disks, a kind of rectangular box, two kinds of cubes) to an arranged area and inserting sticks into holes or turning over cloths. The maximum STEF score is 100 points. The methods of determining the score and speed of manipulation of each object are described elsewhere [19].

### Reproducibility of the measurements of the upper limb

Prior to conducting this study, the reproducibility of the functional measurements for the upper limb was evaluated using intra-class correlation analysis in 37 patients with stroke. The intra-class correlation coefficients between the first and second measurements performed by two different evaluators within 3 days were as follows: *r* = 0.98 (*p* < 0.01) for the BRS of the upper limb; *r* = 0.99 (*p* < 0.01) for the BRS of the hand; and *r* = 0.99 (*p* < 0.01) for the STEF score.

Two occupational therapists served as evaluators throughout the study. The occupational therapist who treated a patient did not serve as the evaluator for that patient.

### Data analysis

It was investigated whether the RFEs improved the isolation from synergy and ability of manipulating the objects in the hemiplegic upper limb and hand of patients with stroke regardless of whether they were in RFE group 1 or 2 and determined whether statistically significant improvements were seen over the two 2-week RFE and CR sessions in all patients (that is, during the combined 2-week RFE sessions or the combined 2-week CR sessions). Data analyses were performed using the Wilcoxon non-parametric test for the BRS and STEF score and Student's *t*-test for age and duration after onset.

Only *p*-values < 0.20 are reported and *p*-values < 0.05 were considered to be statistically significant.

## Results

### Characteristics of the subjects

The characteristics of the stroke patients in RFE group 1 and RFE group 2 are shown in Table I. The background characteristics, such as age, duration after onset and severity of hemiplegia in the affected upper limb and fingers of RFE group 2 were similar to those of RFE group 1.

### Improvements in isolation from synergy in hemiplegic upper limb

**Figure 4:**
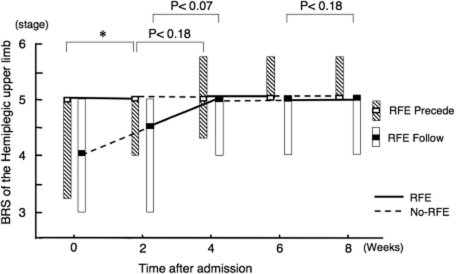
Improvements in isolation from synergy in hemiplegic upper limb by RFE. Changes in the BRS of the hemiplegic upper limb during the study are shown. Data are shown as the median (and quartiles). In RFE group 1, RFE was administered in weeks 1, 2, 5 and 6 and CR was administered in weeks 3, 4, 7 and 8. In RFE group 2, RFE was administered in weeks 3, 4, 7 and 8 and CR was administered in weeks 1, 2, 5 and 6. RFE group 1 is indicated by striped columns, and RFE group 2 is indicated by open columns. Thick line indicates RFE session, and broken line indicates CR session. RFE group 2 is indicated by open columns and broken line. *p < 0.05. Abbreviations: RFE, repetitive facilitation exercise; BRS, Brunnstrom stage.

RFE group 1 underwent RFE sessions in weeks 1, 2, 5 and 6 (Figure 4). In this group, the median (and quartiles) BRS of the upper limb significantly improved from 5.0 (3.3–5.0) at week 0 to 5.0 (4.0–5.0) at the end of the first 2-week RFE session (*p* < 0.05), whereas it showed a non-significant trend for improvement during the subsequent 2-week CR session (*p* = 0.18). RFE group 2 did not show a significant improvement in the median (and quartiles) BRS during the first CR session and showed a non-significant trend for improvement in the subsequent 2-week RFE session from 4.5 (3.0–5.0) to 5.0 (4.0–5.0) (*p* = 0.07).

RFE group 1 showed no further improvements during the second 2-week RFE session (weeks 5 and 6) or the subsequent CR session. RFE group 2 showed a tendency to improve only in the second 2-week RFE session, although the improvement was not statistically significant (*p* = 0.18).

There was no significant difference in the degree of improvement seen over the 8-week period between RFE group 1 and RFE group 2.

### Improvements in isolation from synergy in hemiplegic hand

**Figure 5:**
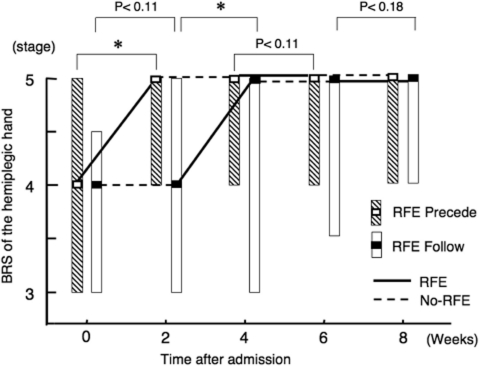
Improvements in isolation from synergy in hemiplegic hand by RFE. Data are shown as the median (and quartiles). Two 2-week RFE sessions were administered interspersed by two 2-week CR sessions. *p < 0.05. Abbreviation: BRS, Brunnstrom stage.

RFE group 1 showed a statistically significant improvement in the median (and quartiles) BRS of the hemiplegic hand from 4.0 (3.0–5.0) to 5.0 (4.0–5.0) in the first 2-week RFE session (*p* < 0.05), but showed no significant improvement during the subsequent CR session (Figure 5). RFE group 2 showed a non-significant trend for improvement during the first CR session (*p* = 0.11) and a significant improvement during the first RFE session (*p* <0.05).

During the second RFE and CR sessions, both RFE group 1 and RFE group 2 showed a nonsignificant trend for improvement only in the 2-week RFE session (*p* = 0.11 and *p* = 0.18, respectively).

There was no difference in the degree of improvement in the BRS of the hand seen over the 8-week study period between RFE group 1 and RFE group 2.

### Improvement in ability of manipulating the objects by hemiplegic upper limb

**Figure 6:**
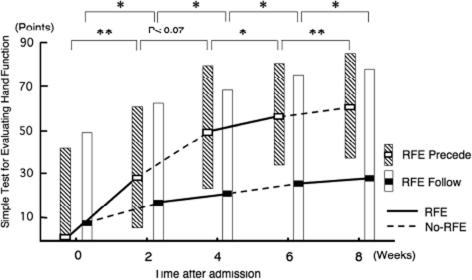
Improvement in ability of manipulating the objects by hemiplegic upper limb by RFE. Data are shown as the median (and quartiles). Two 2-week RFE sessions were administered interspersed by two 2-week CR sessions. *p < 0.05, **p < 0.01. Abbreviation: STEF, Simple Test for Evaluating Hand Function.

RFE group 1 showed a significant improvement (*p* < 0.01) in the STEF score during the first 2-week RFE session and a non-significant trend for improvement (*p* = 0.07) during the subsequent 2-week CR session (Figure 6). RFE group 2 showed statistically significant improvements in the STEF score during both the first 2-week CR session (*p* < 0.05) and the subsequent 2-week RFE session (*p* <0.05).

During the second RFE and CR sessions, RFE group 1 showed significant improvement in the STEF score during both the second RFE and the second CR sessions (*p* <0.05 and *p* < 0.01, respectively). RFE group 2 showed further significant improvement both in the RFE session (*p* < 0.05) and in the CR session (*p* < 0.05).

The median (and quartiles) improvement in the STEF score over the 8-week period in RFE group 1 and RFE group 2 was 35 (31–44) and 2 (0–26), respectively. The difference between the two groups was statistically significant (*p* <0.01).

### Improvements in isolation from synergy and ability of manipulating the objects of hemiplegic upper limb during combined first and second 2-week sessions of RFE or CR in all patients

**Figure 7:**
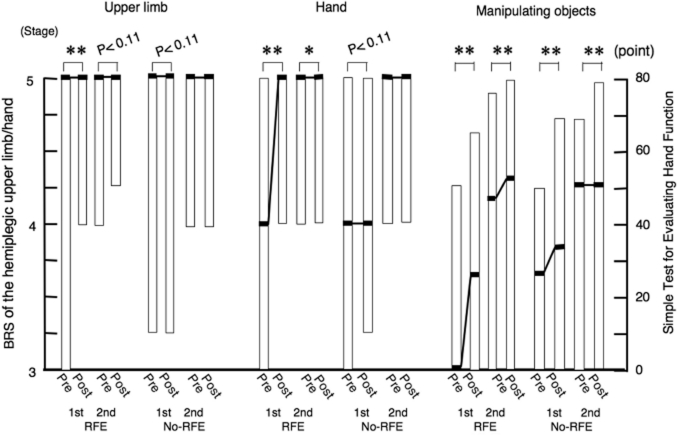
Improvements in isolation from synergy of the hemiplegic upper limb and hand and the ability of manipulating objects during 2-week sessions of RFE or CR in all patients. Data for the BRS of the hemiplegic upper limb and hand and the STEF of the upper limb in the first and second 2-week sessions of RFE or CR among all 23 patients were combined. Data are shown as the median (and quartiles). Pre denotes the beginning of the indicated session. Post denotes the end of the indicated session. *p < 0.05, **p < 0.01. Abbreviations: BRS, Brunnstrom stage; STEF, Simple Test for Evaluating Hand Function.

Figure 7 shows the combined data of the 11 patients in RFE group 1 and the 12 patients in RFE group 2. The improvement in the BRS of the hemiplegic upper limb was statistically significant during the first 2-week RFE session (*p* <0.01), but did not reach non-significant trend for improvement during the first 2-week CR session (*p* = 0.11). There was a non-significant trend (*p* = 0.11) for improvement during the second 2-week RFE session, but no improvement during the second 2-week CR session. The BRS of the hand significantly improved during both the first 2-week RFE session (*p* <0.01) and the second 2-week RFE session (*p* < 0.05). There was a non-significant trend for improvement during the first 2-week CR session (*p* = 0.11), but no improvement during the second 2-week CR session.

The STEF score significantly improved during both the first 2-week RFE session (*p* < 0.01) and the second 2-week RFE session (*p* <0.01), as well as during both the first 2-week CR session (*p* <0.01) and the second 2-week CR session (*p* < 0.01).

## Discussion

The effects of the RFEs used in the novel facilitation technique that was developed on the isolation from synergy and the manipulation of objects of the hemiplegic upper limb were examined in 23 patients with stroke. Improvements in isolation from synergy of the upper limb and hand, as assessed by the BRS, were seen during the 2-week RFE sessions, although some did not reach statistical significance. Improvements in manipulating the objects of hemiplegic upper limb were seen in both the RFE sessions and the CR sessions in both groups.

Although there were no differences between RFE group 1 and RFE group 2 in the degree of improvement of the BRS of the upper limb and hand over the 8-week period, the former group showed greater improvement of the STEF score than the latter. The combined improvement in isolation from synergy seen during the two 2-week RFE sessions in all 23 patients was statistically significant, while that seen during the two CR sessions did not reach statistical significance.

This study employed a cross-over design across individuals to exclude heterogeneity between subjects and to compare the functional improvements during RFE and CR sessions performed in different orders. The influences of the aetiology and lesion site on functional improvement were not analysed because of the small number of subjects and the similarity in lesion site due to the selection criteria employed.

The novel facilitation methods used in this study were designed to give focused sufficient physical stimulation to realize the patient's intended movements. These physical stimulation were the stretch reflex, skin-muscle reflex, and α–γ linkage, which were induced by tapping or rubbing the muscles, rapid passive stretching of the muscle or slight resistance against the intended movements. Although these stimulations used in these methods are similar to current neurophysiological facilitation techniques, these stimulations were given sufficiently strongly to induce the target movements when synchronized or through temporal combinations and postures of the stretched muscles. These methods allowed direct elicitation of isolated movements of each finger, wrist and shoulder and combined movements of the shoulder, elbow, wrist and fingers, which were similar to the PNF technique but differed from it in the proximal stimulations used to elicit movements. These methods also attached greater importance to proximal movements than to distal movements and to shoulder flexion-adduction than to shoulder extension-abduction. In particular, these facilitation methods could be repeated so smoothly that it was possible to perform as many as 500–800 repetitions (that is 100 repetitions of each of five-to-eight patterns) within 30 minutes.

The effects of the interventions on the hemiplegic upper limb and hand were evaluated using the BRS, which reflects the degree of isolation from synergy, and the STEF score [19, 20], which reflects the ability or performance of manipulating the objects by the hemiplegic upper limb as evaluated by the speed of manipulation of objects. This study did not evaluate the patients' disabilities using scales such as the ADL because these measurements, when related to the upper limb, mainly reflect compensation by the non-hemiplegic side [21].

In contrast to the small improvements in the motor function of the hemiplegic upper limb achieved using neurophysiological facilitation techniques, repetitive training of complex hand and arm movements [22] or an early increased-intensity interdisciplinary upper limb therapy programme following acute stroke [23] and increased intensity of neurophysiotherapy [5, 24], thermal stimulation to the hemiplegic upper limb, facilitating its movements, promoted both recovery in sensory disturbance and isolation from synergy in acute stroke patients [11]. Neurophysiological studies suggested that repetition of identical movements is crucial for motor learning [25] and repetitive elicitation of voluntary movements free from synergy led to the isolation from synergy in the hemiplegic lower limb [18].

These results suggest that the RFEs using the novel facilitation technique involving sufficient repetitive elicitation of identical voluntary movements of the upper limb and fingers are beneficial for improvements in isolation from synergy and manipulating objects.

It is unclear whether the improvements seen during the RFE sessions were due to the intervention or to spontaneous/intrinsic recovery following stroke. However, it is reasonable to expect that the earlier therapy is started, regardless of whether it involves CR or RFEs, the greater the neurological improvement will be, as the duration after stroke onset is inversely related to the degree of neurological recovery [26].

In the Copenhagen stroke study, the best possible upper extremity function was achieved by 80% of the patients within 3 weeks after stroke onset and by 95% within 9 weeks [27]. Many of these subjects were expected to show little functional improvement during the 2-month experimental period, especially within the 2-week sessions, because the BRS of the hemiplegic upper limb in the subjects at 15.6 ± 16.8 weeks (mean±SD) after onset was 4.0 (3.0–5.0) (median, quartiles) in the upper limb and 4.0 (3.0–5.0) in the hand.

In the present study, the treatments were started more than 1 month after onset; furthermore, the second 2-week RFE and CR sessions were carried out more than 2 months after onset. RFE group 2 showed greater improvements in the BRS of the upper limb and hand during the first RFE session, which was started after the first 2-week CR session, than during the preceding CR session. In the second RFE session, the improvements were thought to be due to the intervention, because significant spontaneous/intrinsic recovery following stroke was not expected.

In the combined second 2-week RFE or CR sessions in both groups, the improvement in the BRS of the hand was statistically significant only during the 2-week RFE session (*p* < 0.05) and little improvement was seen during the 2-week CR session. This result indicates the effectiveness of the RFEs for the functional recovery of hemiplegia.

An aspect of the RFEs that might have played a role in promoting motor functional improvement is the elicitation of voluntary movements of the hemiplegic limb. The early recovery of voluntary movements induced by the RFEs may have resulted in increased use of the hemiplegic upper limb and hand during voluntary training performed by the patients themselves.

In terms of intensity, the typical constraint-induced movement therapy (CIMT) [28] in which the patient performs tasks using the paretic upper extremity for 6 hours is much harder for patients than RFEs performed for 20–30 minutes. Repetitive realization of the patient's intended movements using RFEs to strengthen the related neural circuits would be more crucial than the intensity of the treatment; in the CIMT, the patient recruits many types of movements of the hemiplegic upper limb including many non-targeted movements that differ from the patient's intended movements, as in tria-l-and-error tests.

Additionally, if a subject selects and enforces objects that he/she had targeted after numerous trial-and-error tests in contrast to the error-free elicitation of targeted movements of the fingers, it might hinder the functional recovery of isolated finger movements. In any case, motor functional recovery of a hemiplegic finger requires more repetitive voluntary upper limb and finger movements that are isolated from synergy by facilitation techniques, because it is difficult for patients to move the hemiplegic upper limb alone, especially the fingers. RFEs for the hemiplegic upper limb improved the functional recovery when voluntary movements were elicited by the novel facilitation technique, although patients who did not show the effects of this facilitation might gain little from RFEs.

Recent studies have shown that brain plasticity contributes to functional recovery from paralysis. Forced use of the hemiplegic hand after brain damage in adult primates [29] and finger tracking training in adult humans who suffered a stroke [30] led to changes in motor representation in the primary motor cortex. TMS studies showed that repetitive motor action by facilitation techniques [31] and CIMT [32] increased the excitability in the motor cortex. Although the effects of repetition of voluntary movements elicited by the new RFEs on motor representation in the primary motor cortex or excitability in the motor cortex were not examined in this study, repetition of voluntary movements elicited by the RFEs might influence the plasticity of the brain.

Further research is needed to confirm the effectiveness of RFE using the novel facilitation technique on functional recovery of the hemiplegic upper limb by a randomized control study and the effect of RFEs on plasticity of the brain, such as the motor representation in the primary motor cortex or excitability in the motor cortex, by a fMRI or TMS study.

## References

[1] Kott MBS, Voss DB (1956). Proprioceptive neuromuscular facilitation.

[2] Brunnstrom S (1970). Movement therapy in hemiplegia: A neuro-physiological approach.

[3] Bobath B (1978). Adult hemiplegia: Evaluation and treatment.

[4] Kraft GH, Fitts SS, Hammond MC (1992). Techniques to improve function of the arm and hand in chronic hemiplegia. Archives of Physical Medicine and Rehabilitation.

[5] Lincoln NB, Parry RH, Vass CD (1999). Randomized, controlledtrial to evaluate increased intensity of physiotherapy treatment of arm function after stroke. Stroke.

[6] Page SJ, Sisto S, Levine P, Johnston MV, Hughes M (2001). Modified constraint induced therapy: A randomized feasibility and efficacy study. Journal of Rehabilitation Research and Development.

[7] Page S, Levine Peter (2003). Forced use after TBI: Promoting plasticity and function through practice. Brain Injury.

[8] Hesse S, Werner C, Pohl M, Rueckriem S, Mehrholz J, Lingnau ML (2005). Computerized arm training improves the motor control of the severely affected arm after stroke: A single-blinded randomized trial in two centers. Stroke.

[9] Feys H, De Weerdt W, Verbeke G, Steck GC, Capiau C, Kiekens C, Dejaeger E, Van Hoydonck G, Vermeersch G, Cras P (2004). Early and repetitive stimulation of the arm can substantially improve the long-term outcome after stroke: A 5-year follow-up study of a randomized trial. Stroke.

[10] Takeuchi N, Chuma T, Matsuo Y, Watanabe I, Ikoma K (2005). Repetitive transcranial magnetic stimulation of contralesional primary motor cortex improves hand function after stroke. Stroke.

[11] Chen JC, Liang CC, Shaw FZ (2005). Facilitation of sensory and motor recovery by thermal intervention for the hemiplegic upper limb in acute stroke patients: A single-blind randomized clinical trial. Stroke.

[12] Platz T, Eickhof C, van Kaick S, Engel U, Pinkowski C, Kalok S, Pause M (2005). Impairment-oriented training or Bobath therapy for severe arm paresis after stroke: A single-blind, multicentre randomized controlled trial. Clinical Rehabilitation.

[13] Dickstein R, Hocherman S, Pillar T, Shaham R (1986). Stroke rehabilitation. Three exercise therapy approaches. Physical Therapy.

[14] Luke C, Dodd KJ, Brock K (2004). Outcomes of the Bobath concept on upper limb recovery following stroke. Clinical Rehabilitation.

[15] Van Peppen RP, Kwakkel G, Wood-Dauphinee S, Hendriks HJ, Van der Wees PJ, Dekker J (2004). The impact of physical therapy on functional outcomes after stroke: What's the evidence?. Clinical Rehabilitation.

[16] Antonov I, Antonova I, Kandel ER, Hawkins R (2003). Activity-dependent presynaptic facilitation and hebbian LTP are both required and interact during classical conditioning in Aplysia. Neuron.

[17] Butefisch C, Hummelsheim H, Denzler P, Mauritz K-H (1995). Repetitive training of isolated movements improves the outcome of motor rehabilitation of the centrally paretic hand. Journal of the Neurological Sciences.

[18] Kawahira K, Shimodozono M, Ogata A, Tanaka N (2004). Addition of intensive repetition of facilitation exercise to multidisciplinary rehabilitation promotes motor functional recovery of the hemiplegic lower limb. Journal of Rehabilitation Medicine.

[19] Kawahira K, Noma T, Iiyama J, Etoh S, Ogata A, Shimodozono M (2009). Improvements in limb kinetic apraxia by repetition of a newly designed facilitation exercise in a patient with corticobasal degeneration. International Journal of Rehabilitation Research.

[20] Yamanaka H, Kawahira K, Arima M, Shimodozono M, Etoh S, Tanaka N, Tsujio S (2005). Evaluation of skilled arm movements in patients with stroke using a computerized motor-skill analyzer for the arm. The International Journal of Rehabilitation Research.

[21] Feys H, De Weerdt W, Verbeke G, Steck GC, Capiau C, Kiekens C, Dejaeger E, Van Hoydonck G, Vermeersch G, Cras P (2004). Early and repetitive stimulation of the arm can substantially improve the long-term outcome after stroke: A 5-year follow-up study of a randomized trial. Stroke.

[22] Woldag H, Waldmann G, Heuschkel G, Hummelsheim H (2003). Is the repetitive training of complex hand and arm movements beneficial for motor recovery in stroke patients?. Clinical Rehabilitation.

[23] Rodgers H, Mackintosh J, Price C, Wood R, McNamee P, Fearon T, Marritt A, Curless R (2003). Does an early increased-intensity interdisciplinary upper limb therapy programme following acute stroke improve outcome?. Clinical Rehabilitation.

[24] Langhammer B, Stanghelle JK (2000). Bobath or motor relearning programme? A comparison of two different approaches of physiotherapy in stroke rehabilitation: A randomized controlled study. Clinical Rehabilitation.

[25] Asanuma H, Keller A (1991). A neuronal mechanisms of motor learning in mammals. NeuroReport.

[26] Jørgensen HS, Nakayama H, Raaschou HO, Vive-Larsen J, Støier M, Olsen TS (1995). Outcome and time course of recovery in stroke. Part II: Time course of recovery. The Copenhagen stroke study. Archives of Physical Medicine and Rehabilitation.

[27] Nakayama H, Jørgensen HS, Raaschou HO, Olsen TS (1994). Recovery of upper extremity function in stroke patients: The Copenhagen Stroke Study. Archives of Physical Medicine and Rehabilitation.

[28] Blanton S, Wolf SL (1999). An application of upper-extremity constraint-induced movement therapy in a patient with subacute stroke. Physical Therapy.

[29] Nudo RJ, Wise BM, SiFuentes F, Millken GW (1996). Neural substrates for the effects of rehabilitative training on motor recovery after ischemic infarct. Science.

[30] Carey JR, Kimberley TJ, Lewis SM, Auerbach EJ, Dorsey L, Rundquist P, Ugurbil K (2002). Analysis of fMRI and finger tracking training in subjects with chronic stroke. Brain.

[31] Hummelsheim H, Hauptmann B, Neumann S (1995). Influence of physiotherapeutic facilitation techniques on motor evoked potentials in centrally paretic hand extensor muscles. Electroencephalography and Clinical Neurophysiology.

[32] Liepert J, Bauder H, Wolfgang HR, Miltner WH, Taub E, Weiller C (2000). Treatment-induced cortical reorganization after stroke in humans. Stroke.

